# 1-Hy­droxy-11*H*-benzo[*b*]fluoren-11-one

**DOI:** 10.1107/S160053681104760X

**Published:** 2011-12-03

**Authors:** Kew-Yu Chen, Ming-Jen Chang, Tzu-Chien Fang

**Affiliations:** a Department of Chemical Engineering, Feng Chia University, 40724 Taichung, Taiwan

## Abstract

The title compound, C_17_H_10_O_2_, is nearly planar, the maximum atomic deviation being 0.053 (2) Å. In the mol­ecule, an intra­molecular O—H⋯O hydrogen bond generates an *S*(6) ring motif. In the crystal, inversion-related mol­ecules are linked by pairs of weak C—H⋯O hydrogen bonds, forming dimers. π–π stacking is observed in the crystal structure, the closest centroid–centroid distance being 3.7846 (16) Å.

## Related literature

For the spectroscopy and preparation of the title compound, see: Aquino *et al.* (2005 ▶); Tang *et al.* (2011 ▶). For applications of proton-transfer dyes, see: Chen & Pang (2009 ▶, 2010 ▶); Chuang *et al.* (2011 ▶); Han *et al.* (2010 ▶); Ito *et al.* (2011 ▶); Jung *et al.* (2009 ▶); Lim *et al.* (2011 ▶). For related structures, see: Chen *et al.* (2011*a* ▶,*b* ▶); Li *et al.* (2007 ▶); Saeed & Bolte (2007 ▶). For graph-set theory, see: Bernstein *et al.* (1995 ▶).
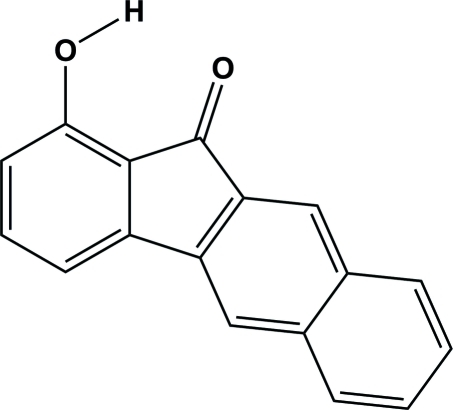


## Experimental

### 

#### Crystal data

C_17_H_10_O_2_
*M* *_r_* = 246.25
Monoclinic, 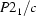

*a* = 12.474 (2) Å
*b* = 6.4401 (12) Å
*c* = 15.601 (3) Å
β = 109.188 (3)°
*V* = 1183.6 (4) Å^3^
*Z* = 4
Mo *K*α radiation
μ = 0.09 mm^−1^
*T* = 297 K
0.42 × 0.22 × 0.12 mm

#### Data collection

Bruker SMART 1000 CCD area-detector diffractometer
6331 measured reflections
2310 independent reflections
1322 reflections with *I* > 2σ(*I*)
*R*_int_ = 0.087

#### Refinement

*R*[*F* ^2^ > 2σ(*F* ^2^)] = 0.047
*wR*(*F* ^2^) = 0.153
*S* = 1.03
2310 reflections
176 parameters
H atoms treated by a mixture of independent and constrained refinement
Δρ_max_ = 0.19 e Å^−3^
Δρ_min_ = −0.16 e Å^−3^

### 

Data collection: *SMART* (Bruker, 2007 ▶); cell refinement: *SAINT* (Bruker, 2007 ▶); data reduction: *SAINT*; program(s) used to solve structure: *SHELXS97* (Sheldrick, 2008 ▶); program(s) used to refine structure: *SHELXL97* (Sheldrick, 2008 ▶); molecular graphics: *ORTEP-3 for Windows* (Farrugia, 1997 ▶); software used to prepare material for publication: *WinGX* (Farrugia, 1999 ▶).

## Supplementary Material

Crystal structure: contains datablock(s) I, global. DOI: 10.1107/S160053681104760X/xu5365sup1.cif

Structure factors: contains datablock(s) I. DOI: 10.1107/S160053681104760X/xu5365Isup2.hkl

Supplementary material file. DOI: 10.1107/S160053681104760X/xu5365Isup3.cml

Additional supplementary materials:  crystallographic information; 3D view; checkCIF report

## Figures and Tables

**Table 1:** Hydrogen-bond geometry (Å, °)

*D*—H⋯*A*	*D*—H	H⋯*A*	*D*⋯*A*	*D*—H⋯*A*
O2—H2*A*⋯O1	1.11 (4)	1.90 (4)	2.877 (3)	145 (3)
C3—H3*A*⋯O1^i^	0.93	2.52	3.369 (3)	151

Symmetry code: (i) 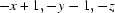.

**Fig. 1.**
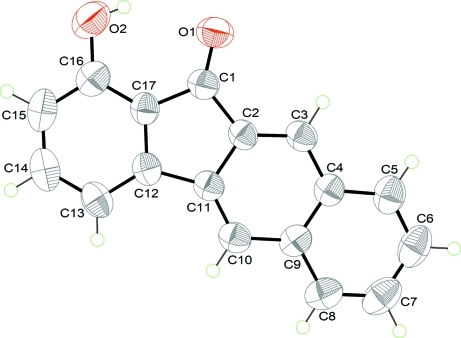
The molecular structure of the title compound, showing 50% probability displacement ellipsoids.

**Fig. 2.**
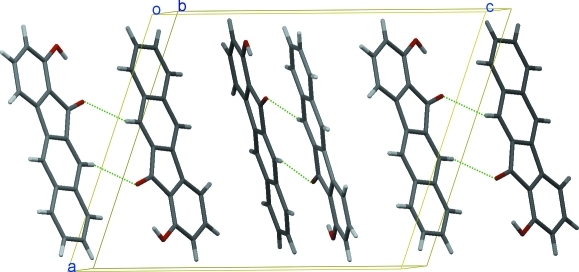
A section of the crystal packing of the title compound, viewed down the b axis. Green dashed lines denote the intermolecular C—H···O hydrogen bonds.

## supplementary crystallographic information

### Comment

The excited-state intramolecular proton transfer (ESIPT) reaction of 7-hydroxy-1-indanone and its derivatives has been investigated for past years (Aquino *et al.*, 2005; Tang *et al.*, 2011), which incorporates transfer of a hydroxy proton to the carbonyl oxygen through a intramolecular six-membered-ring hydrogen-bonding system. The unusual photophysical property of the resulting proton-transfer tautomer has found many important applications (Chen *et al.*, 2009, 2010; Lim *et al.*, 2011; Ito *et al.*, 2011; Han *et al.*, 2010; Jung *et al.*, 2009).

The molecular structure of the title compound (HBO) comprises a 7-hydroxy-1-indanone unit having a naphthalene ring fused on one side (Figure 1). The molecule is nearly planar, which is consistent with previous studies (Chen *et al.*, 2011a; Li *et al.*, 2007; Saeed *et al.*, 2007). HBO possesses an intramolecular O—H···O hydrogen bond (Table 1), which generates an S(6) ring motif (Chen *et al.*, 2011b). In the crystal (Figure 2), inversion-related molceules are linked by a pair of weak C—H···O hydrogen bonds (Table 1), forming a cyclic dimers with *R*_2_^2^(10) graph-set motif (Bernstein *et al.*, 1995). π—π stacking is observed between the tetracyclic plane and its adjacent one, the closest centroid-centroid distance being 3.7846 (16) Å [symmetry code: 1 - *x*, -*y*, 1 - *z*].

### Experimental

The title compound was synthesized according to the literature (Tang *et al.*, 2011). Yellow needle-shaped crystals suitable for the crystallographic studies reported here were isolated over a period of six weeks by slow evaporation from the chloroform solution.

### Refinement

Hydroxy H atom was located in a Fourier map and refined isotropically. Other H atoms ware placed geometrically and refined using a riding model with C—H = 0.93 Å and *U*_iso_(H) = 1.2*U*_eq_(C).

### Crystal data

**Table:**

C_17_H_10_O_2_	*F*(000) = 512
*M**_r_* = 246.25	*D*_x_ = 1.382 Mg m^−^^3^
Monoclinic, *P*2_1_/*c*	Mo *K*α radiation, λ = 0.71073 Å
Hall symbol: -p 2ybc	Cell parameters from 1918 reflections
*a* = 12.474 (2) Å	θ = 2.7–24.7°
*b* = 6.4401 (12) Å	µ = 0.09 mm^−^^1^
*c* = 15.601 (3) Å	*T* = 297 K
β = 109.188 (3)°	Parallelepiped, yellow
*V* = 1183.6 (4) Å^3^	0.42 × 0.22 × 0.12 mm
*Z* = 4	

### Data collection

**Table:**

Bruker SMART 1000 CCD area-detector diffractometer	1322 reflections with *I* > 2σ(*I*)
Radiation source: fine-focus sealed tube	*R*_int_ = 0.087
graphite	θ_max_ = 26.0°, θ_min_ = 1.7°
φ and ω scans	*h* = −14→15
6331 measured reflections	*k* = −7→7
2310 independent reflections	*l* = −19→16

### Refinement

**Table:**

Refinement on *F*^2^	Primary atom site location: structure-invariant direct methods
Least-squares matrix: full	Secondary atom site location: difference Fourier map
*R*[*F*^2^ > 2σ(*F*^2^)] = 0.047	Hydrogen site location: inferred from neighbouring sites
*wR*(*F*^2^) = 0.153	H atoms treated by a mixture of independent and constrained refinement
*S* = 1.03	*w* = 1/[σ^2^(*F*_o_^2^) + (0.0642*P*)^2^ + 0.2285*P*] where *P* = (*F*_o_^2^ + 2*F*_c_^2^)/3
2310 reflections	(Δ/σ)_max_ < 0.001
176 parameters	Δρ_max_ = 0.19 e Å^−^^3^
0 restraints	Δρ_min_ = −0.16 e Å^−^^3^

### Special details

**Table:**

Geometry. All e.s.d.'s (except the e.s.d. in the dihedral angle between two l.s. planes) are estimated using the full covariance matrix. The cell e.s.d.'s are taken into account individually in the estimation of e.s.d.'s in distances, angles and torsion angles; correlations between e.s.d.'s in cell parameters are only used when they are defined by crystal symmetry. An approximate (isotropic) treatment of cell e.s.d.'s is used for estimating e.s.d.'s involving l.s. planes.
Refinement. Refinement of *F*^2^ against ALL reflections. The weighted *R*-factor *wR* and goodness of fit *S* are based on *F*^2^, conventional *R*-factors *R* are based on *F*, with *F* set to zero for negative *F*^2^. The threshold expression of *F*^2^ > σ(*F*^2^) is used only for calculating *R*-factors(gt) *etc*. and is not relevant to the choice of reflections for refinement. *R*-factors based on *F*^2^ are statistically about twice as large as those based on *F*, and *R*- factors based on ALL data will be even larger.

### Fractional atomic coordinates and isotropic or equivalent isotropic displacement parameters (Å^2^)

**Table:**

	*x*	*y*	*z*	*U*_iso_*/*U*_eq_	
O1	0.66186 (16)	−0.4250 (3)	0.11797 (12)	0.0730 (6)	
O2	0.88146 (17)	−0.3141 (4)	0.24218 (14)	0.0836 (6)	
H2A	0.816 (3)	−0.412 (6)	0.194 (3)	0.147 (15)*	
C1	0.6346 (2)	−0.2548 (4)	0.14017 (15)	0.0515 (6)	
C2	0.52192 (19)	−0.1511 (3)	0.10894 (14)	0.0466 (6)	
C3	0.4220 (2)	−0.2150 (4)	0.04737 (15)	0.0527 (6)	
H3A	0.4171	−0.3429	0.0187	0.063*	
C4	0.3259 (2)	−0.0847 (4)	0.02760 (14)	0.0505 (6)	
C5	0.2190 (2)	−0.1414 (5)	−0.03430 (16)	0.0656 (7)	
H5A	0.2111	−0.2688	−0.0638	0.079*	
C6	0.1281 (2)	−0.0144 (6)	−0.05160 (18)	0.0764 (9)	
H6A	0.0587	−0.0549	−0.0928	0.092*	
C7	0.1378 (2)	0.1778 (6)	−0.00777 (19)	0.0774 (9)	
H7A	0.0747	0.2637	−0.0195	0.093*	
C8	0.2396 (2)	0.2395 (4)	0.05214 (17)	0.0655 (7)	
H8A	0.2452	0.3682	0.0803	0.079*	
C9	0.33640 (19)	0.1115 (4)	0.07200 (14)	0.0516 (6)	
C10	0.4427 (2)	0.1731 (4)	0.13508 (15)	0.0538 (6)	
H10A	0.4497	0.3015	0.1637	0.065*	
C11	0.53325 (19)	0.0456 (3)	0.15340 (14)	0.0478 (6)	
C12	0.65335 (19)	0.0694 (4)	0.21356 (13)	0.0485 (6)	
C13	0.7106 (2)	0.2252 (4)	0.26996 (16)	0.0631 (7)	
H13A	0.6733	0.3451	0.2778	0.076*	
C14	0.8266 (2)	0.1985 (5)	0.31532 (18)	0.0714 (8)	
H14A	0.8664	0.3039	0.3533	0.086*	
C15	0.8842 (2)	0.0228 (5)	0.30603 (18)	0.0714 (8)	
H15A	0.9617	0.0115	0.3366	0.086*	
C16	0.8262 (2)	−0.1378 (4)	0.25083 (16)	0.0602 (7)	
C17	0.71100 (19)	−0.1116 (4)	0.20486 (14)	0.0490 (6)	

### Atomic displacement parameters (Å^2^)

**Table:**

	*U*^11^	*U*^22^	*U*^33^	*U*^12^	*U*^13^	*U*^23^
O1	0.0818 (13)	0.0622 (12)	0.0679 (12)	0.0176 (10)	0.0151 (10)	−0.0086 (9)
O2	0.0615 (12)	0.1010 (16)	0.0844 (14)	0.0228 (12)	0.0186 (10)	0.0023 (12)
C1	0.0613 (15)	0.0508 (14)	0.0419 (12)	0.0043 (12)	0.0163 (11)	0.0011 (10)
C2	0.0527 (14)	0.0471 (13)	0.0392 (11)	0.0003 (11)	0.0140 (10)	−0.0014 (10)
C3	0.0620 (15)	0.0513 (14)	0.0431 (12)	0.0013 (12)	0.0151 (11)	−0.0029 (10)
C4	0.0537 (14)	0.0595 (15)	0.0379 (11)	0.0019 (11)	0.0146 (10)	0.0028 (10)
C5	0.0568 (16)	0.0860 (19)	0.0495 (14)	−0.0037 (15)	0.0114 (12)	0.0012 (13)
C6	0.0502 (16)	0.116 (3)	0.0570 (16)	0.0029 (17)	0.0098 (13)	0.0123 (17)
C7	0.0581 (18)	0.108 (3)	0.0659 (18)	0.0236 (17)	0.0205 (14)	0.0195 (18)
C8	0.0675 (18)	0.0757 (18)	0.0571 (15)	0.0177 (14)	0.0256 (14)	0.0093 (13)
C9	0.0511 (14)	0.0645 (16)	0.0410 (12)	0.0086 (12)	0.0174 (11)	0.0086 (11)
C10	0.0636 (16)	0.0537 (14)	0.0463 (13)	0.0024 (13)	0.0211 (11)	−0.0014 (11)
C11	0.0539 (14)	0.0511 (14)	0.0402 (12)	0.0016 (11)	0.0177 (10)	0.0006 (10)
C12	0.0541 (14)	0.0552 (14)	0.0371 (11)	−0.0062 (11)	0.0161 (10)	0.0012 (10)
C13	0.0717 (19)	0.0627 (16)	0.0529 (14)	−0.0096 (14)	0.0176 (13)	−0.0059 (12)
C14	0.0688 (19)	0.080 (2)	0.0587 (16)	−0.0256 (16)	0.0121 (14)	−0.0024 (14)
C15	0.0489 (15)	0.101 (2)	0.0588 (16)	−0.0135 (16)	0.0096 (12)	0.0049 (16)
C16	0.0574 (16)	0.0745 (18)	0.0506 (14)	0.0049 (14)	0.0202 (12)	0.0085 (13)
C17	0.0492 (14)	0.0589 (15)	0.0384 (11)	−0.0019 (11)	0.0137 (10)	0.0009 (10)

### Geometric parameters (Å, °)

**Table:**

O1—C1	1.231 (3)	C7—H7A	0.9300
O2—C16	1.358 (3)	C8—C9	1.409 (3)
O2—H2A	1.11 (4)	C8—H8A	0.9300
C1—C17	1.465 (3)	C9—C10	1.423 (3)
C1—C2	1.486 (3)	C10—C11	1.349 (3)
C2—C3	1.364 (3)	C10—H10A	0.9300
C2—C11	1.429 (3)	C11—C12	1.492 (3)
C3—C4	1.412 (3)	C12—C13	1.370 (3)
C3—H3A	0.9300	C12—C17	1.400 (3)
C4—C5	1.413 (3)	C13—C14	1.397 (4)
C4—C9	1.426 (3)	C13—H13A	0.9300
C5—C6	1.352 (4)	C14—C15	1.374 (4)
C5—H5A	0.9300	C14—H14A	0.9300
C6—C7	1.399 (4)	C15—C16	1.387 (4)
C6—H6A	0.9300	C15—H15A	0.9300
C7—C8	1.365 (4)	C16—C17	1.390 (3)
			
C16—O2—H2A	105 (2)	C10—C9—C4	119.9 (2)
O1—C1—C17	125.3 (2)	C8—C9—C4	118.4 (2)
O1—C1—C2	128.8 (2)	C11—C10—C9	120.1 (2)
C17—C1—C2	105.9 (2)	C11—C10—H10A	119.9
C3—C2—C11	122.1 (2)	C9—C10—H10A	119.9
C3—C2—C1	130.0 (2)	C10—C11—C2	119.6 (2)
C11—C2—C1	107.90 (19)	C10—C11—C12	132.0 (2)
C2—C3—C4	119.3 (2)	C2—C11—C12	108.37 (19)
C2—C3—H3A	120.4	C13—C12—C17	119.8 (2)
C4—C3—H3A	120.4	C13—C12—C11	133.1 (2)
C3—C4—C5	122.5 (2)	C17—C12—C11	107.16 (19)
C3—C4—C9	119.0 (2)	C12—C13—C14	118.0 (3)
C5—C4—C9	118.5 (2)	C12—C13—H13A	121.0
C6—C5—C4	121.4 (3)	C14—C13—H13A	121.0
C6—C5—H5A	119.3	C15—C14—C13	122.6 (3)
C4—C5—H5A	119.3	C15—C14—H14A	118.7
C5—C6—C7	120.3 (3)	C13—C14—H14A	118.7
C5—C6—H6A	119.8	C14—C15—C16	119.7 (2)
C7—C6—H6A	119.8	C14—C15—H15A	120.2
C8—C7—C6	120.3 (3)	C16—C15—H15A	120.2
C8—C7—H7A	119.9	O2—C16—C17	121.5 (2)
C6—C7—H7A	119.9	O2—C16—C15	120.5 (2)
C7—C8—C9	121.1 (3)	C17—C16—C15	118.0 (3)
C7—C8—H8A	119.4	C16—C17—C12	121.9 (2)
C9—C8—H8A	119.4	C16—C17—C1	127.4 (2)
C10—C9—C8	121.7 (2)	C12—C17—C1	110.7 (2)
			
O1—C1—C2—C3	−1.4 (4)	C3—C2—C11—C12	−178.2 (2)
C17—C1—C2—C3	178.4 (2)	C1—C2—C11—C12	−0.1 (2)
O1—C1—C2—C11	−179.3 (2)	C10—C11—C12—C13	0.7 (4)
C17—C1—C2—C11	0.5 (2)	C2—C11—C12—C13	179.2 (2)
C11—C2—C3—C4	−1.0 (3)	C10—C11—C12—C17	−179.0 (2)
C1—C2—C3—C4	−178.6 (2)	C2—C11—C12—C17	−0.5 (2)
C2—C3—C4—C5	−179.0 (2)	C17—C12—C13—C14	1.7 (3)
C2—C3—C4—C9	0.7 (3)	C11—C12—C13—C14	−178.0 (2)
C3—C4—C5—C6	179.5 (2)	C12—C13—C14—C15	−0.6 (4)
C9—C4—C5—C6	−0.1 (3)	C13—C14—C15—C16	−1.0 (4)
C4—C5—C6—C7	−0.3 (4)	C14—C15—C16—O2	−178.8 (2)
C5—C6—C7—C8	0.7 (4)	C14—C15—C16—C17	1.5 (4)
C6—C7—C8—C9	−0.7 (4)	O2—C16—C17—C12	179.8 (2)
C7—C8—C9—C10	−179.3 (2)	C15—C16—C17—C12	−0.5 (3)
C7—C8—C9—C4	0.3 (4)	O2—C16—C17—C1	−2.8 (4)
C3—C4—C9—C10	0.0 (3)	C15—C16—C17—C1	176.9 (2)
C5—C4—C9—C10	179.7 (2)	C13—C12—C17—C16	−1.2 (3)
C3—C4—C9—C8	−179.6 (2)	C11—C12—C17—C16	178.5 (2)
C5—C4—C9—C8	0.1 (3)	C13—C12—C17—C1	−178.9 (2)
C8—C9—C10—C11	179.1 (2)	C11—C12—C17—C1	0.8 (2)
C4—C9—C10—C11	−0.4 (3)	O1—C1—C17—C16	1.4 (4)
C9—C10—C11—C2	0.2 (3)	C2—C1—C17—C16	−178.4 (2)
C9—C10—C11—C12	178.5 (2)	O1—C1—C17—C12	179.0 (2)
C3—C2—C11—C10	0.6 (3)	C2—C1—C17—C12	−0.8 (2)
C1—C2—C11—C10	178.7 (2)		

### Hydrogen-bond geometry (Å, °)

**Table:**

*D*—H···*A*	*D*—H	H···*A*	*D*···*A*	*D*—H···*A*
O2—H2A···O1	1.11 (4)	1.90 (4)	2.877 (3)	145 (3)
C3—H3A···O1^i^	0.93	2.52	3.369 (3)	151

Symmetry codes:  (i) −*x*+1, −*y*−1, −*z*.

## References

[bb1] Aquino, A. J. A., Lischka, H. & Hättig, C. (2005). *J. Phys. Chem. A*, **109**, 3201–3208. 10.1021/jp050288k 16833649

[bb2] Bernstein, J., Davis, R. E., Shimoni, L. & Chang, N.-L. (1995). *Angew. Chem. Int. Ed. Engl.* **34**, 1555–1573.

[bb3] Bruker (2007). *SMART* and *SAINT* Bruker AXS Inc., Madison, Wisconsin, USA.

[bb4] Chen, K.-Y., Fang, T.-C. & Chang, M.-J. (2011*a*). *Acta Cryst.* E**67**, o992. 10.1107/S1600536811010956 PMC3099973 21754249

[bb5] Chen, W.-H. & Pang, Y. (2009). *Tetrahedron Lett.* **50**, 6680–6683.

[bb6] Chen, W.-H. & Pang, Y. (2010). *Tetrahedron Lett.* **51**, 1914–1918.

[bb7] Chen, K.-Y., Wen, Y.-S., Fang, T.-C., Chang, Y.-J. & Chang, M.-J. (2011*b*). *Acta Cryst.* E**67**, o927. 10.1107/S1600536811009718 PMC3099748 21754197

[bb8] Chuang, W.-T., Hsieh, C.-C., Lai, C.-H., Lai, C.-H., Shih, C.-W., Chen, K.-Y., Hung, W.-Y., Hsu, Y.-H. & Chou, P.-T. (2011). *J. Org. Chem.* **76**, 8189–8202. 10.1021/jo2012384 21942211

[bb9] Farrugia, L. J. (1997). *J. Appl. Cryst.* **30**, 565.

[bb10] Farrugia, L. J. (1999). *J. Appl. Cryst.* **32**, 837–838.

[bb11] Han, D. Y., Kim, J. M., Kim, J., Jung, H. S., Lee, Y. H., Zhang, J. F. & Kim, J. S. (2010). *Tetrahedron Lett.* **51**, 1947–1951.

[bb12] Ito, Y., Amimoto, K. & Kawato, T. (2011). *Dyes Pigments*, **89**, 319–323.

[bb13] Jung, H. Y., Kim, H. J., Vicens, J. & Kim, J. S. (2009). *Tetrahedron Lett.* **50**, 983–987.

[bb14] Li, Z., Xu, J.-H., Rosli, M. M. & Fun, H.-K. (2007). *Acta Cryst.* E**63**, o3435.

[bb15] Lim, C.-K., Seo, J., Kim, S., Kwon, I. C., Ahn, C.-H. & Park, S. Y. (2011). *Dyes Pigments*, **90**, 284–289.

[bb16] Saeed, A. & Bolte, M. (2007). *Acta Cryst.* E**63**, o2757.

[bb17] Sheldrick, G. M. (2008). *Acta Cryst.* A**64**, 112–122. 10.1107/S0108767307043930 18156677

[bb18] Tang, K.-C., Chang, M.-J., Lin, T.-Y., Pan, H.-A., Fang, T.-C., Chen, K.-Y., Hung, W.-Y., Hsu, Y.-H. & Chou, P.-T. (2011). *J. Am. Chem. Soc.* **133**, 17738–17745. 10.1021/ja2062693 21957929

